# Predictors of In-Hospital Mortality Among Stroke Patients in Mashhad, Iran: A Novel Circular Approach to Incorporate Spatial Component

**DOI:** 10.34172/jrhs.11685

**Published:** 2026-02-21

**Authors:** Nooshin Akbari Sharak, Morteza Najibi, Mohammad Taghi Shakeri

**Affiliations:** ^1^Department of Biostatistics, School of Health, Mashhad University of Medical Sciences, Mashhad, Iran; ^2^Student Research Committee, Department of Biostatistics, School of Health, Mashhad University of Medical Sciences, Mashhad, Iran; ^3^Rheumatology, Department of Clinical Sciences, Lund University and Skåne University Hospital, Lund, Sweden; ^4^Social Determinants of Health Research Center, Mashhad University of Medical Sciences, Mashhad, Iran

**Keywords:** Stroke, In-hospital mortality, Circular data, Emergency medical services, Bearing angle

## Abstract

**Background:**

Stroke remains a global health challenge, with its burden disproportionately affecting developing nations, including Iran. Rapid access to medical care is crucial for improving outcomes. However, spatial and temporal factors often leads to delays, adversely impacting survival. This study investigated predictors of in-hospital mortality among stroke patients in Mashhad, Iran, with a novel focus on spatial directionality using circular statistical methods.

**Study Design:**

A retrospective cohort study.

**Methods:**

The data of 1,171 stroke patients transported to Ghaem Hospital (2018–2019) were analyzed in this study. Pre-hospital delays, demographics, and clinical factors were assessed alongside spatial directionality, represented by the bearing angle between patients’ residences and the hospital. Circular logistic regression was used to model in-hospital mortality, incorporating both linear and circular predictors.

**Results:**

The in-hospital mortality rate was 14.3%. Independent predictors included age (OR: 1.03, 95% CI: 1.01–1.04), length of stay (OR: 1.02, 95% CI: 1.01–1.04), triage level (OR: 2.31, 95% CI: 1.20–4.45), ambulance accessibility (OR: 0.97, 95% CI: 0.96–0.99), and the sine of the bearing angle (OR: 1.37, 95% CI: 1.02–1.83). Mortality was higher along the north-south axis, potentially reflecting disparities in healthcare access and population characteristics. Gender and final diagnosis were not significant predictors.

**Conclusion:**

Overall, age, length of stay, triage level, ambulance accessibility, and spatial directionality were significant predictors of in-hospital stroke mortality. The circular statistical approach provided added value by detecting directional disparities not captured through conventional methods, underscoring the need for spatially informed interventions to reduce inequities in stroke outcomes.

## Background

 Stroke is a serious global health challenge, with its burden on public health increasing over time.^1^ In addition, it is the second leading cause of death and disability among adults worldwide.^2^ Despite its largely preventable nature, nearly two-thirds of all stroke-related deaths occur in developing countries.^3-5^ In Iran, the in-hospital mortality rate has been reported to be 18.71% for stroke patients. This mortality rate increases in 1-month and 1-year periods, thereby increasing by one-third in a 1-year mortality rate.^6^

 Identifying risk factors for stroke-related mortality is critical to improving prevention strategies while reducing its incidence.^7^ One key factor is timely access to advanced medical care, as stroke management requires urgent intervention.^8^ Studies have shown that every minute of delay in treating ischemic stroke results in the loss of approximately 1.9 million brain cells, emphasizing the importance of rapid treatment.^9^ While both pre-hospital and in-hospital delays influence outcomes, pre-hospital delay, the time from symptom onset to hospital arrival, is frequently longer and has a greater impact on prognosis. Emergency medical services (EMS) play a crucial role in minimizing pre-hospital delays while optimizing stroke care.^10^

 Moreover, patient residence and accessibility to healthcare facilities are significant determinants of stroke outcomes. Research highlights that neighborhood characteristics (e.g., environmental and socio-economic factors) can influence stroke incidence and mortality.^11^ Geographic proximity to hospitals and the distance patients must travel for treatment are crucial in determining delays in admission and subsequent outcomes.^12-14^ For instance, individuals living farther from healthcare facilities are more likely to experience longer delays, increasing their risk of poor outcomes.^14^ These findings underscore the importance of accounting for spatial and accessibility factors in health research.

 In this context, incorporating spatial information in stroke research can provide valuable insights into factors influencing health outcomes. Generally, data can be categorized intolinear and circulartypes. Linear data (e.g., income, age, or weight) are commonly encountered in research. In contrast, circular data arise when measurements are periodic or directional (e.g., time, angles, or compass directions). Circular data are characterized by their cyclical nature, where the starting and ending points coincide.^15^ For example, time measured on a 24-hour clock can be converted into angular data and represented as circular, with values expressed in degrees (0°–360°) or radians (0–2 π).^16-18^

 In addition, circular data are widely used in various disciplines, including meteorology (e.g., wind directions), biology (e.g., animal movement), physics (e.g., angular motion), and medicine (e.g., circadian rhythms).^19^ This broad applicability underscores the potential of circular data to provide new insights into spatial aspects of stroke outcomes, enhancing our understanding of how directional factors impact health. In this study, a novel application of circular data in stroke research is introduced by incorporating patients’ residential locations relative to the hospital as a circular variable. Specifically, the study focuses on computing the bearing angle, which represents the directional relationship between a patient’s residence and the hospital. By integrating this circular measure with traditional linear predictors, the study aims to determine how spatial relationships affect health outcomes, such as admission delays and mortality.

 By treating the bearing angle as a circular covariate, this study also accounts for directional spatial variability in stroke outcomes. Specifically, the bearing angle can help identify whether patients residing in particular directions relative to the hospital experience different outcomes, which may be influenced by a number of factors, such as road infrastructure, traffic patterns, or environmental barriers that vary by direction.

 To investigate these factors, acircular logistic regression model is applied, which is designed to describe the relationship between a binary response variable (e.g., in-hospital mortality) and circular predictors, alongside linear predictors. This method allows us to evaluate the combined effects of spatial and temporal factors on in-hospital mortality among stroke patients in Mashhad, Iran.

## Methods

###  Study area and data sources

 This retrospective cohort study was conducted in Mashhad, the capital of Razavi Khorasan province in northeastern Iran. It is the second most populous city in the country, with an estimated population of approximately 3.8 million (Statistical Center of Iran). The city operates 79 ambulance vehicles across 59 stations and has 25 public hospitals providing medical care.^20^

 The study evaluated patients with stroke symptoms who were transferred to Ghaem Hospital, a tertiary neurological referral center in eastern Iran, by the EMS between April 2018 and March 2019. It should be noted that this hospital serves as the primary facility for neurology emergencies in the region.^21^ In this study, all methods were performed in accordance with relevant guidelines and regulations, and pre-hospital EMS data and in-hospital data were collected based on the aim of the study. Pre-hospital information was obtained from the EMS system database and included delay time, response time, transport time, revealed access time, and patient location. Delay time indicates the interval between receiving an emergency call and dispatching an ambulance, and response time is the interval between receiving the call and ambulance arrival at the scene. Moreover, transport time implies the duration of patient transport from the scene to the hospital, and revealed access time denotes the sum of response and transport times. Furthermore, patient location demonstrates the geographic coordinates of the caller’s address.

 In-hospital information was retrieved from the hospital’s Health Information System and included patient demographic and clinical details: age and gender, screening time (hour), triage level, length of stay (LOS) in the hospital, hypertension diagnosis, final stroke diagnosis based on ICD-10 codes (I63.0 to I63.9 and I69.4), and in-hospital mortality (the primary study endpoint). Further, pre-hospital data were linked with in-hospital data using emergency mission IDs, ensuring a comprehensive dataset. Additionally, the accessibility rate of ambulances (number of ambulances per one million inhabitants) for each district was calculated.^22^

 To model spatial relationships, patient residential addresses at the time of admission were geocoded to latitude and longitude using Google Maps. These geographic coordinates were then converted into bearing angles relative to Ghaem Hospital, representing the directional spatial relationship between the patient’s location and the hospital. This transformation allowed us to incorporate the bearing angle as a circular covariate in the analysis, thereby enabling the evaluation of how the directional component of spatial accessibility influences in-hospital mortality. It is noteworthy that patients residing outside Mashhad were excluded from the study to ensure consistency in geographic coverage. Missing data were minimal, and a complete-case analysis was performed; records with missing values on any study variable were excluded from the analysis.

###  Statistical analysis

 The normality of quantitative variables was assessed using the Kolmogorov-Smirnov test. Continuous variables were summarized as means ± standard deviations (SD) or medians with interquartile ranges (IQR), depending on their distribution. In addition, quantitative variables were compared using the independent samples t-test for normally distributed data, and associations between categorical variables were evaluated using the chi-square test. The means and SDs of the bearing angle were calculated using circular statistical methods, and the Watson-Williams test was applied to compare mean bearing angles between the two groups.

 The circular mean $(\bar{\theta )}$ and circular SD were computed based on the following formulas^19^:


$$\bar{\theta }=Arctan\left(\frac{\sum_{i=1}^{n}\text{sin}\left(\theta_{i}\right)}{n},\;\frac{\sum_{i=1}^{n}\text{cos}\left(\theta_{i}\right)}{n}\right)$$

where *θ_i_* represents each angular observation, and *n* is the total number of observations.

 The circular SD is calculated as:


$$circular\;SD=\sqrt{-2\ln\left(R\right)}$$

where *R* denotes the mean resultant length, defined as:


$$R=\;\sqrt{{\left(\frac{\sum_{i=1}^{n}\text{sin}\left(\theta_{i}\right)}{n}\right)}^{2}+{\left(\frac{\sum_{i=1}^{n}\text{cos}\left(\theta_{i}\right)}{n}\right)}^{2}}$$

For analytical modeling, univariate logistic regression models were applied to linear variables. Variables with *P* < 0.20 were then included in the final multiple regression model.^23^ Subsequently, a logistic regression model for circular data was employed to identify the risk factors (linear and circular) associated with in-hospital stroke mortality.

 The logistic regression model for circular data aims to describe the relationship between a binary response variable and circular predictors. Consider a binary outcome variable *η *∈ {0,1} that depends on a circular explanatory variable *u* ∈ [0,2π]. The probability of a success, π(*β,* u) is modeled using the binomial circular logistic regression equation as follows:


$$\pi \left(\beta ,\;u\right)=\;\frac{\text{exp}\left\{\beta_{0}+\beta_{1}\cos u+\beta_{2}\sin u\right\}}{1+\exp\left\{\beta_{0}+\beta_{1}\cos u+\beta_{2}\sin u\right\}}$$

where β = (*β*0,*β*1,*β*2)^T^ ∈ R^3^ is the model parameter vector. This formulation incorporates the circular nature of the predictor. The circular variable *u* in our model corresponds exclusively to the spatial bearing angle. In addition, the circular logistic component models the effect of this spatial directional variable, while other non-circular predictors (e.g., age, LOS, and triage level) have been incorporated as linear covariates in the full regression model.

 For *n* independent observations divided into I groups, *i* = 1,2,…,I, each containing *n_i_* observations $\left(n=\overset{I}{\underset{i=1}{\sum }}n_{i}\right)$ . Moreover, the covariates and the number of successes are denoted by *ui* and *νi*, respectively. Considering that these observations follow a binomial distribution, the likelihood function is expressed as^24^:


$$L\left(\beta ,\nu ,u\;\right)=\overset{I}{\underset{i=1}{\prod }}\left(\begin{array}{c} n_{i}\\ \nu_{i} \end{array}\right)\pi {\left(\beta ,\;u_{i}\right)}^{\nu_{i}}{\left(1-\pi \left(\beta ,u_{i}\right)\right)}^{n_{i}-\nu_{i}}$$

The circular logistic regression model, first proposed by Al-Daffaie and Khan,^19^ is an extension of the classical logistic regression model for linear data introduced by Berkson.^25^

 For the fitting of the circular logistic regression model, the function “glm” from the package “CircStats”^26^ is used in R software, version 4.0.2.^27^

 To assess global spatial autocorrelation in in-hospital mortality, Moran’s I was calculated using a hexagonal lattice constructed from geocoded patient locations.

## Results

 In this study, 1,171 patients with stroke symptoms were analyzed, of whom 14.3% (167 patients) experienced in-hospital mortality. Among the cohort, 587 (50.10%) were male with a mean age of 69.92 ± 13.61 years, while 584 (49.90%) were female with a mean age of 70.15 ± 13.93 years. The median of LOS was 3 days (IQR = 6), and 77.20% (904 patients) were discharged within the first week of admission. Table 1 provides a summary of the demographic and EMS characteristics of the study population.

**Table 1:** Demographic and clinical characteristics of patients with symptoms of stroke

**Categorical Variables**	**Total (N=1171)**		**Yes (n=167)**		**No (n=1004)**		***P*** ** value**
	**Number**	**Percent**	**Number**	**Percent**	**Number**	**Percent**	
Gender							0.100
Male	587	50.10	74	44.30	513	51.10	
Female	584	49.90	93	55.70	491	48.90	
Age group (y)							0.001
≤ 60	283	24.20	24	14.40	259	25.80	
> 60	888	75.80	143	85.60	745	74.20	
Residency							0.740
Urban	985	84.10	139	83.20	846	84.30	
Suburban	186	15.90	28	16.80	158	15.70	
Length of stay							0.001
≤ 7	904	77.20	94	56.30	810	80.70	
> 7	267	22.80	73	43.70	194	19.30	
Triage level							0.001
Levels 1 & 2	809	69.10	143	85.60	666	66.30	
Levels 3 & 4	362	30.90	24	14.40	338	33.70	
Final stroke diagnosis							0.001
Yes	299	25.50	60	35.90	239	23.80	
No	872	74.50	107	64.10	765	76.20	
**Continuous variables**	**Mean**	**SD**	**Mean**	**SD**	**Mean**	**SD**	***P *** **value**
Age, mean ± SD	70.00	13.80	73.9 0	13.70	69.40	13.60	0.001
Accessibility rate of the ambulance (per one million inhabitants)	27.30	6.73	26.4	6.73	27.50	7.10	0.060
Delay time (s)	37.30	29.70	42.70	29.91	36.80	29.71	0.040
Response time (min)	9.00	3.90	9.00	4.10	9.00	3.82	0.970
Transport time (min)	21.50	11.90	22.00	13.90	21.50	11.60	0.120
Revealed access (min)	30.50	13.10	31.00	15.11	30.40	12.82	0.610
Screening time (h)	0.25	0.30	0.20	0.10	0.30	0.31	0.040
Distance to the hospital (km)	5.90	2.90	6.00	2.90	5.90	2.90	0.530
LOS, median (IQR)	3.00	6.00	6.00	10.00	2.00	6.00	0.001

*Note*. SD: Standard deviation; LOS: Length of stay; IQR: Interquartile range; LOS was summarized using median (IQR).

*Based on the results, no significant* difference was observed between males and females in terms of in-hospital mortality (50.10% vs. 49.9%, *P* = 0.3). A majority (75.80%) of patients were older than 60 years, and 85.60% of the deaths occurred within this age group (*P* = 0.001). Furthermore, delay time (*P* = 0.040) and LOS (*P* < 0.001) demonstrated statistically significant differences concerning mortality outcomes. Additionally, mortality was noticeably associated with triage level (*P* < 0.001) and final stroke diagnosis (*P* = 0.001). However, the mean values of other variables, including response time, transport time, revealed access, ambulance accessibility rate, and distance to the hospital, did not considerably differ between patients who survived and those who did not (*P* > 0.05).

 The bearing angle, a circular variable, was analyzed using circular statistical methods. The mean bearing angle for the entire cohort was 81.06° ± 54.93°. When stratified by mortality, the bearing angles were 81.47° and 80.01° for survivors and non-survivors, respectively, with no statistically significant difference between the two groups based on the Watson-Williams test (*P* = 0.858).

 The univariate binary logistic regression analysis identified several variables significantly associated with in-hospital mortality among patients with stroke symptoms. They included age, final stroke diagnosis, triage level, screening time, and LOS. Variables with a *P* value < 0.200 in the univariate analysis (e.g., gender, age, final stroke diagnosis, delay time, triage level, screening time, ambulance accessibility rate, LOS, and bearing angle) were subsequently included in the final multivariable logistic regression model.

 The results from the multiple circular logistic regression model revealed that several variables had a statistically significant association with in-hospital mortality among patients with stroke symptoms. They included age (OR = 1.03, 95% CI: 1.01–1.04), LOS (OR = 1.03, 95% CI: 1.01–1.04), triage level (OR = 2.31, 95% CI: 1.45–3.69), accessibility rate of ambulances (OR = 0.97, 95% CI: 0.95–0.99), and the sine of the bearing angle (OR = 1.37, 95% CI: 1.02–1.86).

 After adjusting for other variables in the model, age was positively associated with in-hospital mortality, with the odds increasing by 3% for each additional year. Similarly, LOS was positively associated with in-hospital mortality, with the odds increasing by 3% for each additional day of hospital stay. In addition, triage level showed a strong positive association, with patients assigned higher triage levels having 2.31 times the odds of in-hospital mortality compared to those with lower triage levels. The accessibility rate of ambulances was negatively associated with in-hospital mortality, as the odds decreased by 2% for each unit increase in the accessibility rate of ambulances per one million residents.

 Other variables, including gender, delay time, and final stroke diagnosis, represented no statistically significant associations with in-hospital mortality (*P* > 0.05, Table 2).

**Table 2:** Determining risk factors associated with in-hospital mortality in patients with symptoms of stroke using the circular logistic regression model

**Variables (Reference)**	**OR (95% CI)**	***P*** ** value**
Male/female	1.23 (0.87, 1.74)	0.234
Age (year)	1.03 (1.01, 1.04)	0.001
Final stroke diagnosis (Yes/No)	1.41 (0.97, 2.04)	0.072
Triage level (3 & 4/1 & 2)	2.31 (1.45, 3.69)	0.001
Accessibility rate of ambulance (per one million residents)	0.97 (0.95, 0.99)	0.047
Delay time (s)	1.00 (0.99, 1.01)	0.300
Screening time (h)	0.26 (0.06, 1.09)	0.067
LOS	1.03 (1.01, 1.04)	0.001
Sin (bearing angle)	1.37 (1.02, 1.86)	0.039
Cos (bearing angle)	1.17 (0.92, 1.49)	0.202

*Note*. OR: Odds ratio; CI: Confidence interval; LOS: Length of stay.

 The hexagon-level aggregation of in-hospital mortality demonstrated a significant positive spatial autocorrelation (Moran’s I = 0.30; Z = 12.87; *P* < 0.001), indicating that mortality outcomes were spatially clustered rather than randomly distributed across Mashhad. This supports the presence of underlying geographical patterns and is consistent with the directional north-south variation identified through circular regression.

## Discussion

 Using circular statistical methods, this study assessed whether the directional distribution of patients relative to the hospital was associated with in-hospital mortality. The use of bearing angles provided a novel approach to capturing spatial directionality, thereby complementing conventional geographic analyses. The finding that the sine component of the bearing angle was significantly associated with mortality suggests a directional gradient along the North-South axis. Such asymmetry may reflect underlying differences between northern and southern areas of Mashhad, including variations in healthcare accessibility, EMS coverage, population density, or sociodemographic characteristics. While circular regression identifies the directional dimension of this disparity, the observed spatial clustering in the Moran’s I analysis further supports the presence of underlying geographical structures in mortality risk.

 In this study, the in-hospital stroke mortality rate was 14.30%, which is higher than that reported in some previous studies,^22,24,25^ likely due to the high proportion of elderly patients (75.80% over 60 years old). However, it was lower than rates observed in other studies.^28-31^ Moreover, age emerged as a critical independent predictor of mortality, with each additional year increasing the odds of death by 3%. This finding aligns with that of previous research, highlighting the strong association between advanced age and stroke mortality.^32-34^

 Additionally, the median LOS was three days, with 22.80% of patients hospitalized for over one week. Longer LOS significantly increased the odds of mortality, with a 2% increase for each additional day of hospitalization. These findings are consistent with the results of some other studies, demonstrating that prolonged hospital stays correlate with higher mortality risk.^35-37^

 Ambulance accessibility also played a pivotal role in patient outcomes. In Mashhad, the mean ambulance accessibility rate was 27 ambulances per one million residents in 2018.^22^ Higher accessibility rates were inversely associated with mortality, highlighting the importance of timely EMS interventions. Previous studies indicated that increased EMS accessibility improves the likelihood of receiving thrombolytic therapy while reducing key time intervals, such as emergency physician response time and neurologist evaluation time.^38-40^

 The directional effect observed in this study complements earlier spatial analyses in Mashhad, such as auto-logistic regression models identifying elevated stroke mortality in suburban and northeastern neighborhoods with socioeconomic disadvantages.^41^ In general, these findings confirm the role of geographic and social determinants in shaping stroke outcomes, underscoring the need for targeted interventions to improve EMS distribution and healthcare access in underserved regions.

 While both studies highlight the spatial determinants of mortality, our analysis uniquely leverages circular statistics to identify directional trends, offering a complementary perspective to neighborhood-level autocovariate findings. Overall, these results emphasize the need for targeted healthcare interventions in socioeconomically disadvantaged areas and improved EMS accessibility in underserved regions.

 Likewise, our findings underline the critical need for equitable healthcare resource allocation. Policymakers should prioritize improving EMS station distribution and healthcare accessibility, particularly in northern and southern areas of Mashhad, to address observed disparities.

 This study had several limitations. It was based on a single year of registry data, limiting the ability to assess temporal trends. In addition, the registry lacked variables such as stroke subtype, severity, comorbidities, and hospital discharge policies, which could influence outcomes. Additionally, operational factors (e.g., hospital crowding, EMS workload, and traffic conditions) were unavailable and could not be incorporated, possibly resulting in residual confounding. Accordingly, future studies incorporating real-time EMS and environmental data may provide a more comprehensive understanding of geographic influences on mortality.

**Highlights**

- Circular statistics were applied to assess directional predictors of stroke mortality.
- In-hospital stroke mortality rate was 14.3% among 1,171 emergency medical service-transported patients.
- Age, length of stay, triage level, and ambulance access predicted mortality risk.
- A significant north-south direction effect indicated spatial healthcare disparities.
- Circular analysis revealed spatial trends that were undetectable by conventional methods.

## Conclusion

 In summary, the findings identified age, length of hospital stays, and ambulance accessibility as significant predictors of in-hospital stroke mortality in Mashhad. Importantly, the use of circular statistical methods introduced a novel way to capture directional disparities, revealing a North–South gradient in mortality that complements conventional spatial approaches. This methodological advancement highlights the potential of circular statistics to enhance spatial health research by identifying patterns that may otherwise remain undetected. Nevertheless, these findings are based on single-center, single-year registry data and should be interpreted with caution accordingly. Future research with larger, multi-year, and multicenter datasets, as well as inclusion of additional clinical covariates, is warranted to validate and extend these results.

## Acknowledgments

 The authors wish to thank Mashhad University of Medical Sciences for their support. They also acknowledge the Medical Stroke Department in Ghaem Hospital for providing access to data.

## Artificial Intelligence Use Disclosure

 In this study, ChatGPT (OpenAI) was used for language editing assistance in preparing this manuscript.

## Competing Interests

 The authors declare they have no competing interests.

## Ethical Approval

 The study protocol was approved by the Ethics Committee of Mashhad University of Medical Sciences (ethical code: IR.MUMS.FHMPM.REC.1403.265). The required data were collected with official authorization from Mashhad University of Medical Sciences (research project No. 981153).

## Funding

 This study was self-funded by the authors and received no external financial support from any funding organization.
